# Tumeur du nerf sciatique: neurinome ou neurofibrome? L'IRM peut-elle faire la différence?

**DOI:** 10.11604/pamj.2019.33.242.18200

**Published:** 2019-07-23

**Authors:** Omar El Aoufir, Moulay Rachid EL Hassani, Mohamed Jidane, Meriem Fikri

**Affiliations:** 1 Service de Neuroradiologie, Hôpital des Spécialités (HSR), CHU Ibn Sina, Faculté de Médecine et de Pharmacie de Rabat, Université Mohammed V, RABAT, Maroc

**Keywords:** Nerf sciatique, tumeurs nerveuses, neurinome, neurofibrome, IRM, Sciatic nerve, nerve tumor, neurinoma, neurofibroma, MRI

## Abstract

Le nerf sciatique est la branche terminale du plexus sacré. La sciatalgie traduit une souffrance des racines nerveuses. La sciatique relève le plus souvent de l'origine dégénérative discale, la pathologie tumorale reste exceptionnelle. Il s'agit d'une tumeur nerveuse, révélée par un syndrome sciatique rebelle au traitement, diagnostiquée à l'IRM, chez un patient de 33 ans. La pathologie tumorale du nerf sciatique est rare, de diagnostic difficile. Les informations fournies par l'IRM sont cruciales car elles conditionnent l'approche chirurgicale.

## Abstract (en)

The sciatic nerve is the terminal branch of the sacral plexus. Sciatalgia is a nerve root pain. In most cases, sciatica originates from degenerative disc disease. Tumor involving the sciatic nerve is extremely rare. We here report the case of a 33-year old patient with nerve tumor detected on MRI performed for drug-resistant sciatica. Tumor involving the sciatic nerve is rare and diagnosis is difficult. MRI data are crucial for establishing an effective surgical approach.

## Introduction

Le nerf sciatique est le plus volumineux nerf de l'organisme humain. De nombreuses lésions peuvent l'affecter, dont la pathologie tumorale. Les tumeurs nerveuses sont des entités rares et mal connues. Le schwannome représente la forme la plus fréquente. L'objectif à travers ce cas clinique, est de trouver des éléments distinctifs entre le neurofibrome et le neurinome. L'imagerie par résonance magnétique (IRM), joue un rôle majeur dans la caractérisation des lésions tumorales, apportant des éléments clés au diagnostic.

## Patient et observation

**Figure 1:**
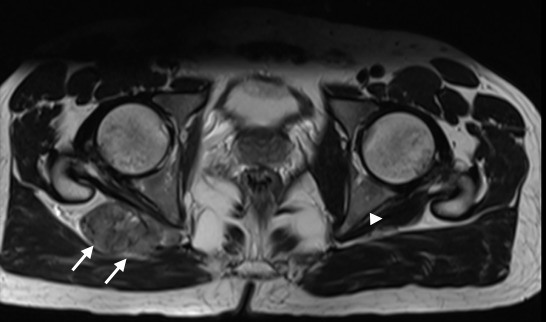
IRM en pondération T2 coupe axiale, montrant un processus lésionnel, centré sur l'échancrure sciatique droite, de signal tissulaire hétérogène, en hypersignal modéré T2 (flèche blanche). La masse soulève le muscle pyramidal droit, aspect normal à gauche (tête de flèche)

**Figure 2:**
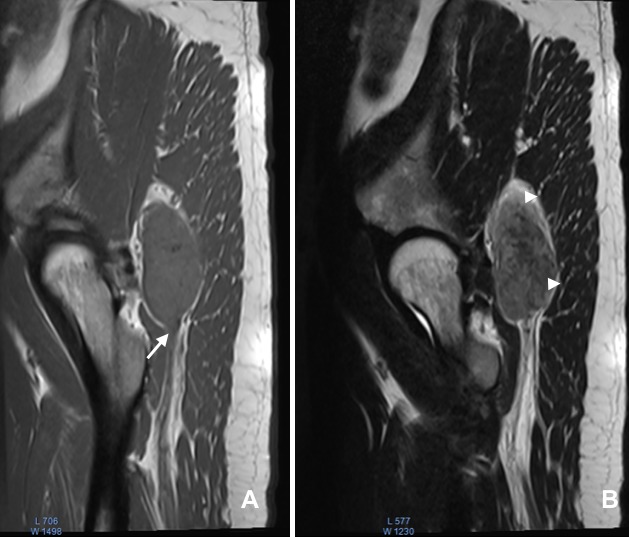
IRM en pondération T1 (A) et T2 (B), en coupes sagittales, montrant un processus lésionnel, de forme fusiforme, développé au dépend du nerf sciatique, le nerf efférent est bien individualisé (flèche blanche). La masse est de densité similaire aux muscles en pondération T1 et en hypersignal modéré hétérogène en pondération T2, elle refoule le muscle grand fessier en arrière

**Figure 3:**
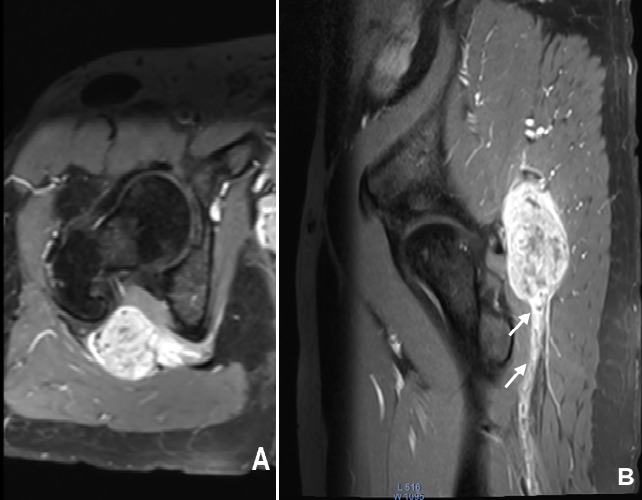
IRM en pondération T1 avec saturation de graisse et injection de Gadolinium, coupe axiale (A) et sagittale (B), tumeur du nerf sciatique rehaussé de façon intense et hétérogène. Notez le rehaussement pathologique de la gaine du nerf sciatique efférent confirmant son infiltration (flèche blanche)

**Figure 4:**
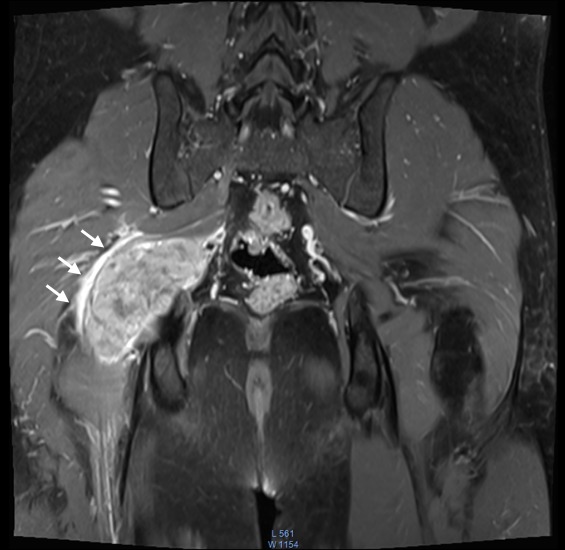
IRM en pondération T2 en saturation de graisse, en coupe coronale, montrant la tumeur centrée sur le nerf sciatique en hyper signal hétérogène. A noter un œdème péri lésionnel en hypersignal (flèche blanche)

Il s'agit d'un patient de 33 ans, sans antécédents, présentant des sciatalgies rebelles au traitement, évoluant depuis 6 mois. L'IRM initiale objective un processus centré sur l'échancrure sciatique droite à cheval sur le canal sous pyramidal, en iso-signal en pondération T1 et en hypersignal modéré hétérogène en pondération T2 (Figure 1, Figure 2). A l'injection de produit de contraste, sur les séquences en saturation de graisse, la masse se rehausse modérément et de façon hétérogène à l'injection de gadolinium, sans envahissement ni infiltration des tissus avoisinants (Figure 3, Figure 4). Le diagnostic retenu est celui d'un neurofibrome. La tumeur est réséquée et l'examen anatomopathologique confirme le diagnostic.

## Discussion

Les tumeurs du nerf sciatique sont souvent de découverte fortuite. Lorsqu'elles sont symptomatiques, elles occasionnent des douleurs et des paresthésies. Les déficits sensitifs ou moteurs sont rares et demeurent tardifs. L'examen clinique cherche une tuméfaction sur le trajet du nerf sciatique, un signe de Tinel évocateur ou des manifestations cutanées témoignant de l'envahissement locorégional. Les examens radiologiques et tout particulièrement l'IRM ont pour rôle de confirmer le diagnostic et d'apprécier l'étendue et l'envahissement. L'analyse minutieuse de la sémiologie radiologique à l'IRM permet dans certains cas, le diagnostic de nature. La traduction radiologique des tumeurs nerveuses dépend de la structure générale du nerf sciatique: un nerf périphérique est constitué d'un ensemble de fascicules au sein desquels se distribuent les fibres nerveuses (l'axone et ses cellules de Schwann satellites). Le fascicule est limité par le périnèvre. L'endonèvre correspond au tissu conjonctif intra fasciculaire. Les fascicules nerveux se rassemblent dans un tissu conjonctif lâche appelé l'épinèvre [1]. Les tumeurs bénignes regroupent les neurinomes ou schwannomes et les neurofibromes. Les tumeurs malignes sont dominées par les sarcomes.

A l'IRM, la stratégie diagnostique repose sur la confirmation de l'origine neurogène de la masse, en la situant sur le trajet nerveux et en repérant le nerf efférent et afférent de part et d'autre du processus tumoral (Figure 2). D'autres signes sont en faveur de l'origine nerveuse: le « fascicular sign » en pondération T2, lorsque la tumeur prend un aspect fasciculé avec des zones de signal intermédiaire et séparés par des zones en hypersignal et le « split-fat-sign » avec persistance d'un liseré graisseux à la périphérie tumorale. Les neurinomes se développent à partir des cellules de Schwann. Ce sont des tumeurs encapsulées, ovoïdes, excentrées par rapport aux troncs nerveux. La forme unifocale reste la plus fréquente [2,3]. En règle générale, elles sont de taille modérée et de croissance lente [4,5]. L'IRM permet d'orienter le diagnostic en mettant en évidence une tumeur de même signal que le tissu musculaire sur les séquences pondérées en T1 et de signal très intense en T2 avec quelques plages centrales en hyposignal [6], se rehaussant le plus souvent de façon intense et homogène. Un &oeligdème péri-lésionnel peut s'associer mais sans envahissement du voisinage. Le neurofibrome, dans sa forme localisée, se traduit à l'IRM comme une tuméfaction fusiforme, de siège central sur le nerf porteur, iso ou légèrement en hypersignal T1, hypersignal T2, avec un rehaussement hétérogène, parfois en cible caractéristique (hyposignal central T2 et une couronne périphérique) (Figure 2, Figure 3). Le neurofibrome Plexiforme est une tumeur nerveuse particulière, infiltrante et tortueuse associée typiquement avec la neurofibromatose type 1. Le contexte clinique et l'aspect étendu et vermiculaire à l'imagerie oriente le diagnostic. Les données de la littérature s'accordent que la nature nerveuse de la tumeur peut-être aisément affirmée [7,8]. La difficulté repose donc sur la différenciation entre schwannome et neurofibrome.

En résumé, le schwannome apparaît plus fréquemment excentré, avec une capsule et des bords bien limités [6,9,10]. La position excentrée de cette tumeur est le seul critère statistique significatif de la présence d'un schwannome [10,11]. Dans notre cas, la position centrale, le signal et le rehaussement hétérogène, nous ont permis de poser le diagnostic de neurofibrome. Les risques de dégénérescence et de récidive du schwannome sont très rares, cela peut être expliqué par l'encapsulation du schwannome [12-13]. Contrairement, le neurofibrome solitaire, non encapsulé, présente un taux de récidive d'environ 23,5% et un taux de dégénérescence maligne, entre 5 et 16%. C'est pourquoi le traitement recommandé pour la résection de la tumeur doit être effectué avec une marge de sécurité [14,15]. Certains auteurs ont proposé une résection en bloc de la tumeur et du tronc porteur, associée à une anastomose épi neurale ou une greffe fasciculaire dans le même temps [16,17]. Pour le patient présenté. Une exérèse complète de la tumeur et une ablation du nerf sciatique ont été pratiquées. L'évolution était favorable.

## Conclusion

Tout syndrome sciatique doit être dument exploré, l'IRM est l'examen de choix, la pathologie tumorale est une étiologie à évoquer. L'approche chirurgicale est différente entre un schwannome et un neurofibrome. Une excision marginale est souvent suffisante pour le neurinome afin de préserver la fonction nerveuse alors que le neurofibrome est considéré comme « inextirpable » et sa résection nécessite certains sacrifices. Le diagnostic de nature est une donnée importante de l'IRM, pour une bonne planification de la stratégie opératoire.

## Conflits d’intérêts

Les auteurs ne déclarent aucun conflit d'intérêts.

## References

[1] Anatoine JC Anatomie et physiologie du nerf périphérique. EMC.

[2] Khouni H, Andrianne R, Nidhal H, Badreddine S, de Leval J, Faouzi MA (2005). Tumeur rétropéritonéale rare: schwannome bénin. Prog Urol.

[3] Kubota Y, Yanai Y, Kumamaru W, Mori Y (2011). Multiple schwannomas in the oral floor: case report. Br J Oral Maxillofac Surg.

[4] Wolock BS, Baugher WH, McCarthy EJ (1989). Neurilemoma of the sciatic nerve mimicking tarsal tunnel syndrome. J Bone Joint Surg Am.

[5] Amezyane T, Pouit B, Bassou D, Lecoules S, Desramé J, Blade JS (2006). Une cause rare de lombosciatique. Rev Med Interne.

[6] Cerofolini E, Landi A, DeSantis G, Maiorana A, Canossi G, Romagnoli R (1991). MR of benign peripheral nerve sheath tumors. J Comput Assist Tomogr.

[7] Park MJ, Seo KN, Kang HJ (2009). Neurological deficit after surgical enucleation of schwannomas of the upper limb. J Bone Joint Surg Br.

[8] Chick G (2010). Tumeurs primitives des nerfs périphériques. EMC - Appar Locomoteur.

[9] Boulanger X, Ledoux JB, Brun AL, Beigelman-Aubry C (2013). Imagerie du plexus brachial non traumatique. J Radiol Diagn Interv.

[10] Bhargava R, Parham DM, Lasater OE, Chari RS, Chen G, Fletcher BD (1997). MR imaging differentiation of benign and malignant peripheral nerve sheath tumors: use of the target sign. Pediatr Radiol.

[11] Beaman FD, Kransdorf MJ, Menke DM (2004). Schwannoma: radiologic-pathologic correlation. Radiographics.

[12] Lollar KW, Pollak N, Liess BD, Miick R, Zitsch RP (2010). Schwannoma of the hard palate. Am J Otolaryngol.

[13] Vartiainen VM1, Siponen M, Salo T, Rosberg J, Apaja-Sarkkinen M (2008). Widening of the inferior alveolar canal: a case report with atypical lymphocytic infiltration of nerve. Oral Surg Oral Med Oral Pathol Oral Radiol Endod.

[14] Martins MD, Taghloubi SA, Bussadori SK, Fernandes KP, Palo RM, Martins MA (2007). Intraosseous schwannoma mimicking a periapical lesion on the adjacent tooth: case report. Int Endod J.

[15] Curtin JP, McCarthy SW (1997). Perineural fibrious thickening within the dental pulp in type 1 neurofibromatosis: a case report. Oral Surg Oral Med Oral Pathol Oral Radiol Endod.

[16] Glicenstein J, Ohana J, Leclercq C (1988). Tumeurs de la main.

[17] Pépin J (1956). Les tumeurs nerveuses des membres.

